# Incidental Findings Suggestive of COVID-19 Pneumonia in Oncologic Patients Undergoing ^18^F-FDG PET/CT Studies: Association Between Metabolic and Structural Lung Changes

**DOI:** 10.2967/jnumed.121.261915

**Published:** 2022-02

**Authors:** Cristina Gamila Wakfie-Corieh, Federico Ferrando-Castagnetto, Alba María Blanes García, Marta García García-Esquinas, Aída Ortega Candil, Cristina Rodríguez Rey, María Nieves Cabrera-Martín, Ana Delgado Cano, José Luis Carreras Delgado

**Affiliations:** 1 Department of Nuclear Medicine, Hospital Clínico San Carlos, Madrid, Spain;; 2 Fundación Para la Investigación Biomédica del Hospital Clínico San Carlos, Madrid, Spain;; 3 Department of Cardiology, Hospital de Clínicas Dr. Manuel Quintela, Montevideo, Uruguay; and; 4 Department of Radiology, Hospital Clínico San Carlos, Madrid, Spain

**Keywords:** COVID-19, pneumonia, lung, cancer, ^18^F-FDG PET/CT

## Abstract

Although the novel coronavirus disease 2019 (COVID-19) can present as nonspecific clinical forms, subclinical cases represent an important route of transmission and a significant source of mortality, mainly in high-risk subpopulations such as cancer patients. A deeper knowledge of the metabolic shift in cells infected with severe acute respiratory syndrome coronavirus 2 could provide new insights about its pathogenic and host response and help to diagnose pulmonary involvement. We explored the potential added diagnostic value of ^18^F-FDG PET/CT scans in asymptomatic cancer patients with suspected COVID-19 pneumonia by investigating the association between metabolic and structural changes in the lung parenchyma. **Methods:** ^18^F-FDG PET/CT studies acquired between February 19 and May 29, 2020, were reviewed to identify those cancer patients with incidental findings suggestive of COVID-19 pneumonia. PET studies were interpreted through qualitative (visual) and semiquantitative (measurement of SUV_max_) analysis evaluating lung findings. Several characteristic signs of COVID-19 pneumonia on CT were described as COVID-19 Reporting and Data System (CO-RADS) categories (*1*–6). After comparing the SUV_max_ of pulmonary infiltrates among different CO-RADS categories, we explored the best potential cutoffs for pulmonary SUV_max_ against CO-RADS categories as the gold standard result to eliminate the possibility that the diagnosis of COVID-19 pneumonia exists. **Results:** On multimodal PET/CT imaging, CT signs classified as CO-RADS category 5 or 6 were found in 16 of 41 (39%) oncologic patients. SUV_max_ was higher in patients with categories 5 and 6 than in patients with category 4 (6.17 ± 0.82 vs. 3.78 ± 0.50, *P* = 0.04) or categories 2 and 3 (3.59 ± 0.41, *P* = 0.01). A specificity of 93.8% (95% CI, 71.7%–99.7%) and an accuracy of 92.9% were obtained when combining a CO-RADS score of 5 or 6 with an SUV_max_ of 2.45 in pulmonary infiltrates. **Conclusion:** In asymptomatic cancer patients, the metabolic activity in lung infiltrates is closely associated with several combined tomographic changes characteristic of COVID-19 pneumonia. Multimodal ^18^F-FDG PET/CT imaging could provide additional information during early diagnosis in selected predisposed patients during the pandemic. The prognostic implications of simultaneous radiologic and molecular findings in cancer patients and other subpopulations at high risk for COVID-19 pneumonia deserve further evaluation in prospective research.

## Visual Abstract

**Figure:**
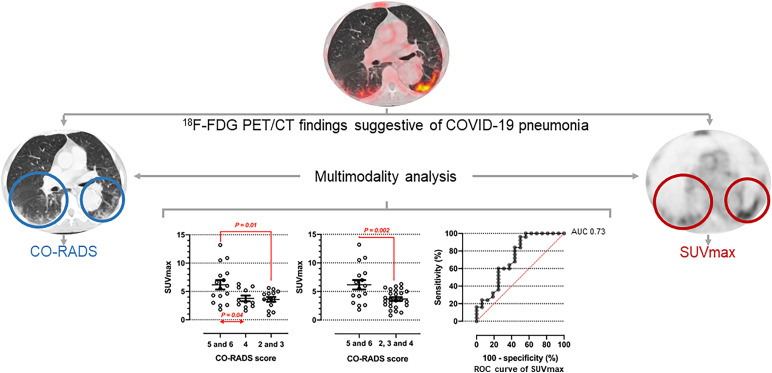

Since December 2019, severe acute respiratory syndrome coronavirus 2 (SARS-CoV-2) has quickly spread worldwide from a cluster of cases in Wuhan, China. Although the novel coronavirus disease 2019 (COVID-19) can present as different, nonspecific clinical forms, subclinical cases represent an important route of transmission and a significant source of morbidity and mortality.

Although COVID-19 is usually confirmed by real-time reverse-transcription polymerase chain reaction (rRT-PCR) in respiratory tract specimens, some imaging techniques may strongly suggest the diagnosis until laboratory results are available. In addition, many concerns have been raised about the low sensitivity of rRT-PCR tests (*1*). In this scenario, chest CT has been positioned as the most useful, noninvasive tool in the diagnosis of COVID-19 pneumonia. Moreover, some CT patterns observed in patients with COVID-19 pneumonia during the pandemic were even more sensitive than rRT-PCR. Despite the limitations of rRT-PCR, it is considered the best diagnostic tool to date.

Several clinical features of the novel infection are particularly challenging in cancer patients. In fact, cancer is a high-risk factor for viral infections, and oncologic patients usually demonstrate an indolent clinical course and a high COVID-19 case fatality rate (*2*,*4*). Unfortunately, differentiation among several respiratory virus and other causes of pneumonitis is difficult in this subpopulation (*5*). So, the rate of suspected infection in asymptomatic predisposed patients should consider the updated epidemiologic data, the risk of infection, and an individualized analytic, imaging, and rRT-PCR or gene sequencing assessment.

Only a few reports and small case series of cancer patients have documented the presence of incidental ^18^F-FDG uptake in the lungs suggestive of COVID-19 pneumonia on PET/CT. These data suggest a potential contribution of this technique to the differential diagnosis of complex or asymptomatic presentations of COVID-19 (*6*–10). In addition, a deeper knowledge of the metabolic shift in cells infected with SARS-CoV-2 could provide new insights about the pathogenesis of viral infection and host response and help to diagnose pulmonary and distant involvement in selected cases. However, a detailed characterization of combined clinical data, radiologic lung findings, and molecular lung findings in cancer patients with COVID-19 pneumonia is still lacking. With our experience, we aimed to explore the potential added diagnostic value of ^18^F-FDG PET/CT scans in asymptomatic cancer patients with suspected COVID-19 pneumonia by investigating the association between metabolic and structural changes in the lung parenchyma.

## MATERIALS AND METHODS

### Study Population

We analyzed 1,065 PET/CT scans acquired from February 19 to May 29, 2020 (Supplemental Figure 1, flowchart; supplemental materials are available at http://jnm.snmjournals.org). After exclusion of all subjects with a non–^18^F-FDG scan, a nononcologic indication, localized brain studies (functional or tumoral brain PET), or symptoms suggestive of respiratory tract infection (e.g., fever, cough, dyspnea, and sneezing), 967 subjects were included. The last exclusion criterion was the result of the updated European Association of Nuclear Medicine recommendations, in which all subjects undergoing PET/CT were questioned to detect any symptoms suggestive of COVID-19 infection or any personal contact with a confirmed case during the last 12–48 h (*11*). The Declaration of Helsinki was respected, and the study was approved by the local Ethical Committee. Because the study was retrospective and took place during the COVID-19 pandemic in Spain, the need for individual informed consent was waived (institutional review board approval 20/524-E).

### Clinical and Analytic Data

To identify patients with incidental findings suggestive of COVID-19 pneumonia, we reviewed the ^18^F-FDG PET/CT data, including clinical and demographic variables, oncologic indication, biochemical profile, and follow-up by clinical and imaging techniques after PET/CT.

### PET/CT Imaging

A usual preparation protocol for ^18^F-FDG PET/CT was followed, acquiring the images after 6 h of fasting in nondiabetic patients or 4 h in diabetic patients with a blood glucose level of less than 200 mg/dL. All patients remained at rest for 40–60 min after intravenous administration of ^18^F-FDG (5 MBq/kg). All studies were acquired following the European Association of Nuclear Medicine guidelines on a Siemens Biograph 6 True Point PET/CT multimodal device with a 6-ring detector CT component, performing diagnostic CT (Topogram Dose Modulation System, CARE Dose4D) with a slice thickness of 5 mm and a reconstruction interval of 3 mm. A first inspirational chest CT study was reconstructed as 2.5-mm slices, at 60 mAs and 110 kV, with a tube rotation time of 0.6 s and a pitch of 1.2. Then, another body CT study was performed from the base of the skull to the mid thigh in the craniocaudal direction, during free breathing. For patients who had not undergone contrast-enhanced CT during the previous month, and in the absence of contraindications such as iodine allergy or kidney failure, an intravenous dose of iodinated contrast medium (130 mL of iohexol, Omnipaque [GE Healthcare], 300 mg I/mL) was administered. In total, 782 of 967 ^18^F-FDG PET/CT studies were performed after iodinated contrast administration. Finally, a PET study was performed at the same locations as the CT study. The acquisition time was 3 min per bed (stretcher) position. Data obtained from PET/CT were merged into a dedicated workstation using the Syngo software system (Siemens Medical Imaging). Regions of interest were manually placed, and SUV_max_ was recorded in the lung parenchyma.

### Image Interpretation and Clinical Referral After PET/CT

PET/CT scans were reviewed by at least a nuclear medicine physician and a radiologist, reaching a consensus on the final interpretation of each study. The pulmonary findings for each PET/CT study were analyzed qualitatively (changes were interpreted as positive when the pulmonary infiltrates showed tracer uptake greater than normal lung activity) and semiquantitatively (SUV_max_). The presence of several diagnostic features in chest CT, such as opacity pattern, bilateralism, lobe involvement, multisegmentation, extension, proximity to visceral pleura and fissures, crazy-paving pattern, hilar lymphadenopathies, and pleural effusion were reported by an expert radiologist. Then, the tomographic changes were combined and characterized applying the COVID-19 Reporting and Data System (CO-RADS) criteria, categorized from 0 to 6 (*12*,*13*). In line with local and regional recommendations, all patients with CO-RADS 4 or 5 COVID-19–suggestive CT findings on PET/CT were sent to the emergency department immediately after contacting the referring oncology team. The emergency and oncology teams determined whether the patient would be admitted to the hospital or would stay in isolation at home, what preventive measures might be used, and what the clinical management plan would be. For patients categorized as CO-RADS 1–3, the PET/CT result was sent to the treating oncologist as usual.

### COVID-19 Diagnosis in the Real-Life Setting

Diagnostic rRT-PCR testing was performed on upper or lower respiratory specimens. When available, one or more serologic criteria were included as a surrogate for rRT-PCR, using a plate-based assay that detects antibodies obtained through enzyme-linked immunosorbent assay of serum from a peripheral vein. A confirmed case of COVID-19 was defined as CO-RADS category 5 or 6 findings (CO-RADS 5 with or without genetic or serologic confirmation by rRT-PCR or enzyme-linked immunosorbent assay techniques, respectively) in the absence of clinical or radiologic findings suggesting other, cancer-related, causes of lung infiltration (radiant or cytostatic pneumonitis, tumoral lymphadenopathies, carcinomatous lymphangitis, or secondary or newly primary tumoral lesions) (*12*). These differential diagnoses were made by at least 1 radiologist and 1 nuclear medicine physician working in consensus. Because of the design and the previously reported false-negative rate of rRT-PCR, the diagnosis could not be confirmed in all subjects in our study sample (*14*,*15*).

### Statistics

Normality of data was determined through Kolmogorov–Smirnoff testing. Continuous data are presented as mean ± SD or as median ± interquartile range (25th–75th percentiles), when appropriate, and discrete or categoric variables are presented as frequencies. Discrete variables were compared through the Fisher exact test. A 1-way ANOVA followed by post hoc testing (Tukey) was applied to compare SUV_max_ among cancer patients with different combined tomographic signs included in the CO-RADS categories, grouped as CO-RADS 5 and 6 versus CO-RADS 4 versus CO-RADS 2 and 3. A second analysis was aimed at categorizing the chest CT pattern as being highly or very highly suggestive of COVID-19 pneumonia (group 1, CO-RADS 5 and 6) versus being suggestive at only an indeterminate or low level (group 2, CO-RADS 2–4). An unpaired *t* test was applied to compare pulmonary SUV_max_ between groups. Because the characteristic chest CT findings included in CO-RADS 5 category have less than optimal sensitivity, we added a second, masked, step to CT interpretation for diagnosis of COVID-19 pneumonia, constructing receiver-operating-characteristic curves to find the best cutoff for pulmonary SUV_max_ against these tomographic criteria (*1*,*12*). With this purpose, sensitivity, specificity, likelihood ratio, and accuracy were calculated for different cutoffs. As the ratio of patients with the disease to patients without the disease does not reflect the true prevalence of the illness, we used the prevalence method to estimate accuracy for each cutoff. Accuracy was calculated considering a local COVID-19 pneumonia prevalence of 1.6% during the study period. A *P* value of less than 0.05 was considered significant (2-tailed). All analyses were performed and graphs created using GraphPad Prism software (version 9.0.0).

## RESULTS

In total, 41 of the 967 patients who underwent ^18^F-FDG PET/CT for oncologic indications during the study period showed pulmonary infiltrates on CT, representing a frequency of 4.2% (41/967).

Lung cancer (*n* = 8), head and neck tumors (*n* = 7), and breast cancer (*n* = 6) were the most frequent oncologic indications for ^18^F-FDG PET/CT, representing 51% (21/41) of patients. A serum biochemical profile was available in 20 of 41 patients, most of them with pulmonary infiltrates categorized as CO-RADS 5 on CT (*P* = 0.004). The clinical and analytic characteristics of the study population are provided in Table 1.

**TABLE 1.** Clinical, Oncologic, and Biochemical Characterization of Study Population

Variable	Group 1 (*n* = 16)	Group 2 (*n* = 25)	*P*
Clinical			
Age (y)	69.8 ± 13.5	64.7 ± 14.9	0.28
Male sex	15 (60.0)	7 (43.8)	0.15
Oncologic			
Cancer staging	5 (12.2)	7 (17.1)	> 0.99
rt-PCR or serologic confirmation*	10 (24.4)	3 (7.3)	0.0014
Biochemical^†^			
Serum C reactive protein (mg/mL)	7.5 ± 8.7	4.4 ± 8.4	0.45
Lymphocyte blood count (per mm^3^)	1.4 ± 0.7	1.2 ± 0.6	0.44
Serum alanine aminotransferase (mg/mL)	18.6 ± 6.5	19.3 ± 4.8	0.82
Serum aspartate aminotransferase (mg/mL)	27.6 ± 12.8	22.3 ± 6.3	0.33
Serum lactate dehydrogenase (mg/mL)	586.1 ± 204.6	473.0 ± 163.8	0.22

*One patient with diagnosis of COVID-19 infection was confirmed by serologic IgG test (enzyme-linked immunosorbent assay).

^†^Analytics were available for patients sent to emergency department with high or very high suspicion based on CO-RADS categories.

Qualitative data are number and percentage; continuous data are mean ± SD.

**FIGURE 1.**
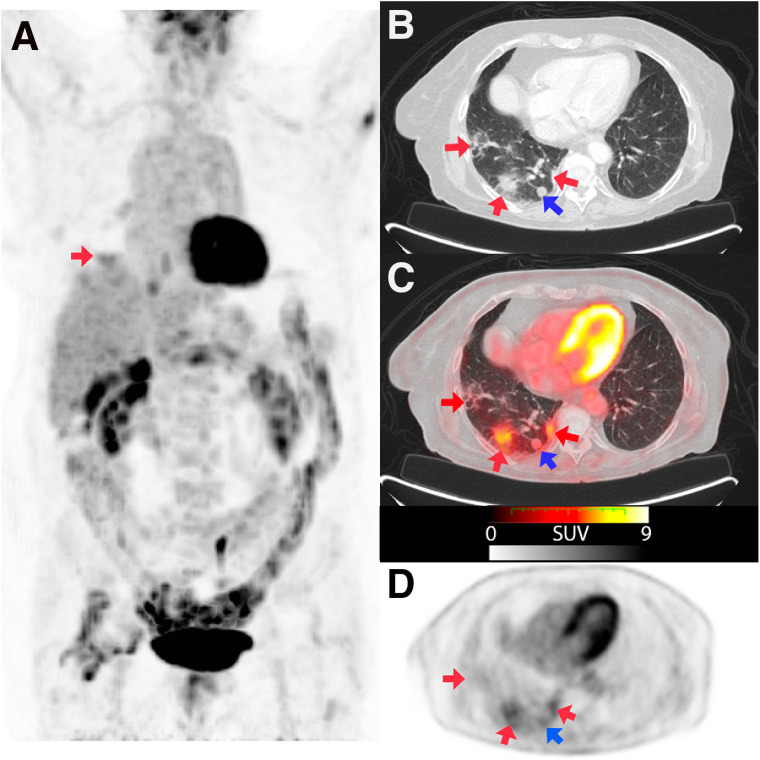
An 86-y-old woman with second primary lung tumor (B and C, blue arrow) referred for PET/CT to assess therapeutic response. Maximum-intensity projection (A) and axial sections with lung window (B) and fusion (C) images showed several pulmonary consolidations located mainly in right inferior lobe (red arrows, CO-RADS 5), with increased ^18^F-FDG uptake (SUV_max_, 4.6). rRT-PCR was positive for COVID-19.

**FIGURE 2.**
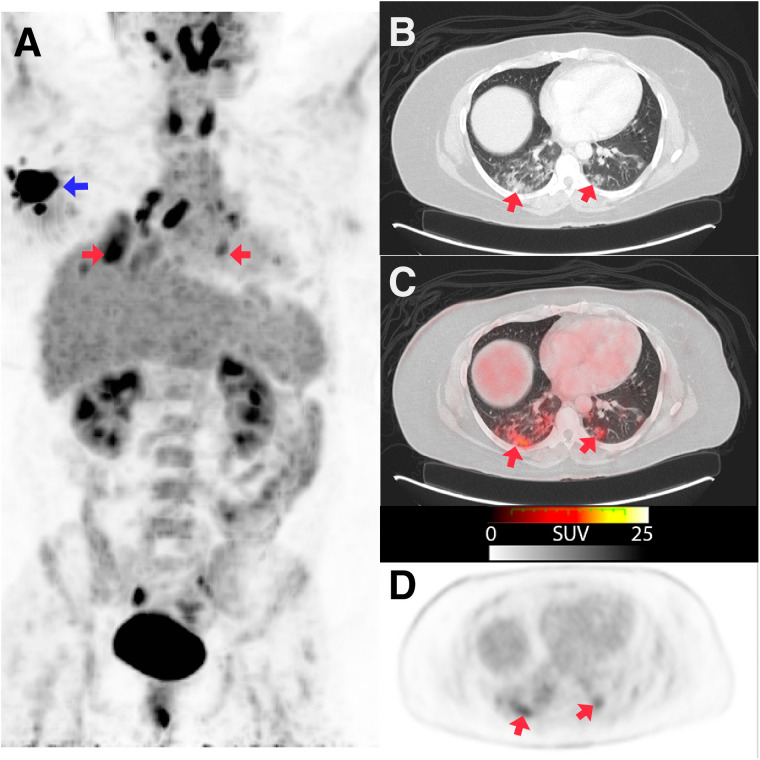
Staging PET/CT of 43-y-old woman with breast cancer (A, blue arrow). Maximum-intensity projection (A) and axial sections with lung window (B) and fusion (C) images show bilateral ground-glass pulmonary infiltrates (red arrows), some of them with pseudonodular morphology, located in both lower lobes and left middle lobe, with diffuse or peripheral distribution (SUV_max_, 7.8). Early rRT-PCR obtained in emergency department was negative for COVID-19, and second test was not available.

CT signs classified as CO-RADS category 5 or 6 were found in 39% (16/41) of our sample. Almost all of this group of patients (15/16, 94%) had ground-glass opacities on CT. Infiltrates were bilateral in 12 patients of this group and peripherally distributed in 10. The presence of subpleural fibrous bands was also frequent (9/16, 56%). A characteristic crazy-paving pattern was detected in 25% (4/16) of the patients. In contrast, no lymphadenopathies or pleural effusion was observed in any subject of this group. These lung changes in 2 patients are shown in Figures 1 and 2.

**FIGURE 3.**
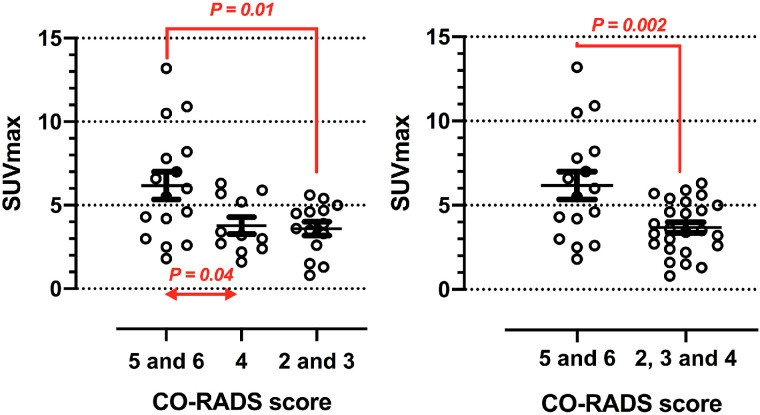
Association of molecular and structural findings observed on multimodal imaging. SUV_max_ is compared among cancer patients of different CO-RADS categories.

Tests for COVID-19 were available for 20 patients; the results were positive in 13 and negative in 7. Twenty-four percent (10/41) were CO-RADS category 6, 14% (6/41) were category 5, 27% (11/41) were category 4, 24% (10/41) were category 3, and 10% (4/41) were category 2. On the qualitative analysis, lung activity was interpreted as positive in 40 patients. After multiple comparisons of SUV_max_ among different CO-RADS categories, a higher SUV_max_ was found for patients with CO-RADS category 5 and 6 than for patients with category 4 (6.17 ± 0.82 vs. 3.78 ± 0.50, *P* = 0.04) or categories 2 and 3 (3.59 ± 0.41, *P* = 0.01) (Fig. 3). After we aggregated tomographic categories, SUV_max_ was higher for group 1 than for group 2 (6.17 ± 0.82 vs. 3.67 ± 0.31, *P* = 0.002) (Fig. 3).

**FIGURE 4.**
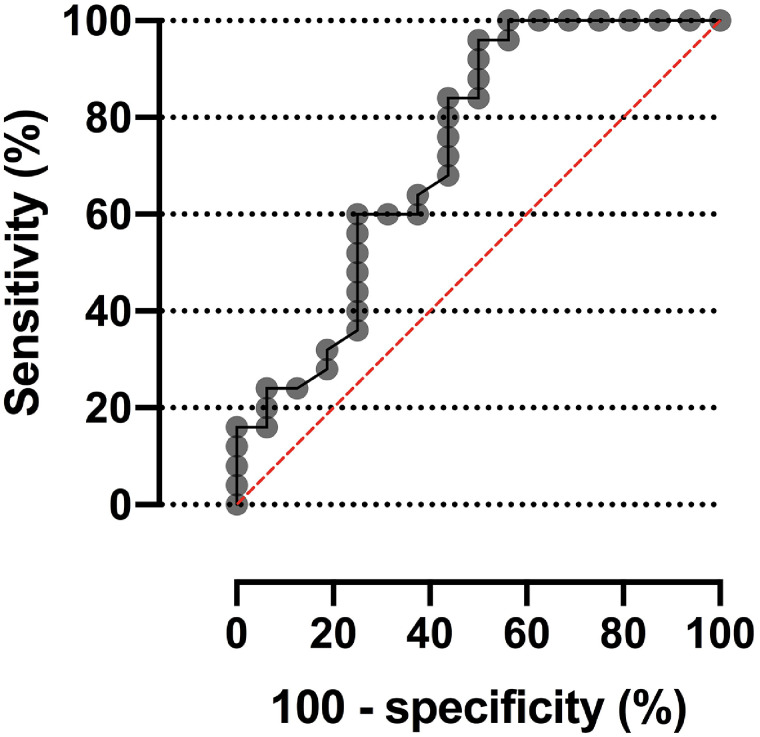
Receiver-operating-characteristic curve of SUV_max_ to detect COVID-19 pneumonia on basis of structural tomographic diagnosis (CO-RADS 5 and 6).

The area under the curve obtained for different cutoffs of SUV_max_ was 0.73 (95% CI, 0.56–0.90; *P* = 0.015) (Fig. 4). The diagnostic yield aiming at eliminating the possibility that the diagnosis exists—estimated by comparing the specificity and likelihood ratio for different SUV_max_ cutoffs against CO-RADS 5 and 6 as a gold standard—is presented in Table 2. An SUV_max_ cutoff of 3.10 obtained a specificity of at least 75.0%. The best specificity (93.8%; 95% CI, 71.7%–99.7%) and a higher likelihood ratio and accuracy were obtained when combining CO-RADS categories 5 and 6 with an SUV_max_ of 2.45 in pulmonary infiltrates.

**TABLE 2.** Potential Usefulness of Different Cutoffs for ^18^F/FDG PET/CT SUV_max_ to Confirm or Eliminate Possibility That Tomographic Diagnosis of COVID-19 Exists

SUV_max_ cutoff	Sensitivity (%)	95% CI (%)	Specificity (%)	95% CI (%)	LR	Accuracy (%)
<2.45	24.0	11.5–43.4	93.8	71.7–99.7	3.8	92.9
<2.55	24.0	11.5–43.4	87.5	64.0–97.8	1.9	86.7
<2.85	32.0	17.2–51.6	81.3	57.0–93.4	1.7	80.7
<3.10	36.0	20.3–55–5	75.0	50.5–89.8	1.4	74.5

LR = likelihood ratio.

A subtotal of 35 of 41 patients with pulmonary infiltrates on ^18^F-FDG studies were followed by imaging techniques during the next few weeks after PET/CT (8 d to 8 mo). Pulmonary findings improved or resolved in 31 of 35 patients, worsened in 2 of 35, and evolved to post–COVID-19 sequelae in 2 of 35. Five patients (5/41) referred from other centers were lost during follow-up. Only 1 patient (1/41), a 78-y-old man with urothelial cancer without signs of recurrence on PET/CT, died after the PET/CT study (8 d afterward, as a result of severe respiratory failure). In this patient, COVID-19 pneumonia was confirmed by rRT-PCR, pulmonary changes were classified as CO-RADS 5 on CT, and SUV_max_ was 6.0 on molecular imaging.

## DISCUSSION

Incidental changes suggesting COVID-19 pneumonia on the chest CT portion of ^18^F-FDG PET/CT studies were found in 4.1% of cancer patients at our center, a lower proportion than previously reported (7.1%–9.2%) (*7*,*8*,*16*). These heterogeneous results could be explained by the differences in health-care facilities and policies in different medical centers and countries, the time-dependent transmissibility of the virus, or other factors related to the study sample (*4*,*11*,*17*).

Although the diagnostic potential of metabolic activity in pulmonary infiltrates of suggestive or confirmed COVID-19 pneumonia on PET/CT has recently been described, the correlation between the CO-RADS scale and the SUV_max_ of the lung parenchyma has not been evaluated in detail until now (*6*–*10*,*16*–*21*). Our preliminary results could provide a new perspective on the pathophysiology of SARS2-CoV-2 lung infection and even redefine the best diagnostic imaging criteria for several patient subpopulations.

The performance of chest CT for the diagnosis of COVID-19 pneumonia could be even better when applying several combined findings as in CO-RADS (*22*). However, the CO-RADS scale has been validated mainly in patients with moderate or severe symptoms and a minor incidence of cancer (21%) (*18*). The specificity obtained through our successive, diagnostic design ranged from 81.2% to 95.5%, considering an SUV_max_ cutoff of 2.25–3.10. Longitudinal investigation has confirmed that a high proportion of asymptomatic patients with COVID-19 pneumonia usually manifest symptoms during the next few days and weeks (*23*). A higher SUV_max_ obtained for those CT findings described as more specific for COVID-19 pneumonia (*9*,*22*) represents a new tool indicating the predictive value of different CO-RADS categories and also suggests its clinical contribution to discarding this viral pneumonia in cancer patients, even before the appearance of symptoms.

The methodologic approach of this study was aimed at exploring whether the SUV_max_ obtained in new pulmonary infiltrates could contribute to eliminating the possibility that the diagnosis of COVID-19 pneumonia exists, that is, improving the specificity obtained through isolated structural changes on chest CT. Not surprisingly, the best specificity was obtained with an SUV_max_ cutoff of 2.25–2.55, a value that has been similarly reported as an indicator of benign etiology. This empiric cutoff and the relationship demonstrated between higher SUV_max_ and more suggestive CO-RADS category agree with observational research on other nonmalignant pulmonary processes (*24*). However, we confirmed that a higher SUV_max_ was observed predominately in the presence of several tomographic signs of COVID-19 pneumonia (Fig. 3). In addition to its diagnostic contribution, the close correlation between structural and metabolic findings could stimulate future research on the pathophysiology of COVID-19 lung injury in predisposed subjects to better characterize the local inflammatory component and its possible changes in response to new therapies.

However, the variation in SUV_max_ observed in lung changes related to COVID-19 pneumonia could also be influenced by factors such as patient weight, motion artifacts, blood glucose levels, dose extravasation, the accuracy of dose calibration, and the time between injection and imaging. Previous reports on certain inflammatory pneumonias found that lung areas with consolidation are associated with a higher SUV_max_ than are areas with ground-glass opacity. Histopathologic examinations have revealed that the number of CD45-positive cells and CD8-positive T lymphocytes in parenchymal lung lesions correlates positively with SUV_max_ (*25*). As it is well known that there are noncellular components in ground-glass opacities (particularly in fluid-filled intraalveolar regions), SUV_max_ is lower in this pulmonary pattern. This supports the hypothesis that the higher the CO-RADS is, the highest will be the SUV_max_, as CO-RADS 5 includes patterns of lung consolidation whereas CO-RADS 3–5 include patterns of ground-glass opacity (*12*,*25*,*26*). In addition, the pulmonary findings related to COVID-19 pneumonia may vary according to the phase of alveolar damage. In the end stage, with fully established fibrosis, the lung parenchyma is destroyed, potentially justifying the lower SUV_max_ found for lung fibrosis (CO-RADS 1) (*12*,*26*). Finally, the pulmonary findings in COVID-19 are not limited to a simple infiltrate of infectious and inflammatory cells but include possible vessel-related damage such as capillary leaks and thrombosis (*26*,*27*). Although these findings are auspicious, the true and complete diagnostic value of different individual CT findings, combined with the local SUV_max_ on ^18^F-FDG PET/CT, should be explored in larger, multicenter experiences.

Several inflammatory lung diseases can be characterized by molecular findings similar to those of COVID-19 pneumonia in cancer patients (*24*). As a consequence, SARS-CoV-2 infection always needs to be distinguished from other viral or bacterial causes of pneumonia, as well as from noninfectious diseases such as pulmonary vasculitis, dermatomyositis, organizing pneumonia and from posttherapeutic changes. Finally, some patients with viral pneumonia may test positively to more than one virus, and the potential lethality of coinfection with SARS-CoV-2 and influenza should not be ignored. In diagnosing COVID-19 pneumonia during a local outbreak, we considered multiple CT findings and expert opinions, and not all patients underwent genetic testing or screening for other sources of infection. Our results should therefore be interpreted cautiously and should be considered as generating only a plausible hypothesis about the value of using PET/CT early to diagnose COVID-19 in cancer patients (*28*).

Our results should encourage all nuclear medicine physicians to pay special attention to incidental ^18^F-FDG PET/CT findings suggesting pneumonia and act as quickly as possible (*29*). During the COVID-19 outbreak in Spain, all nuclear medicine departments followed the safety and prevention protocols provided by the European Association of Nuclear Medicine, in a collective effort to allow for safer diagnosis and treatment procedures (*11*). Importantly, ^18^F-FDG PET/CT is a more complex procedure than chest CT and requires more time, leading a possible increased risk of viral spread. In addition, the probability of diagnosing at least some asymptomatic high-risk patients with COVID-19 infection by various common nuclear medicine procedures such as ^18^F-FDG PET/CT is not negligible (*29*). On the other hand, the oncology team needs to weigh the risk of death and morbidity from COVID-19 against the benefit of applying several therapies. Obtaining more detailed data through PET/CT could have a doubly positive impact by lowering the risk of spreading the infection while increasing the expected benefits of cancer therapy. This complex, critical clinical scenario requires maintaining an adequate balance for each individual.

The main limitation of our study is the small real-life sample of cancer patients with suspected COVID-19 pneumonia and use of only a single center. A second limitation is that the time since infection was not known, preventing us from evaluating the real impact of early diagnosis in predisposed patients. A third limitation is that only 1 examiner interpreted the CT images and that interrater variability in each CO-RADS category could therefore not be evaluated (*22*). Finally, the size of reference bias was not estimated.

## CONCLUSION

In asymptomatic cancer patients, the SUV_max_ of lung parenchyma infiltrates on ^18^F-FDG PET/CT studies is closely associated with several tomographic changes characteristic of COVID-19 pneumonia. Multimodal ^18^F-FDG PET/CT imaging could provide additional information during the diagnosis of COVID-19 in selected patients, even in early stages of the disease. Future prospective experiences are required to define the prognostic value of combining radiologic and molecular findings in cancer patients and other subpopulations at high risk for COVID-19 pneumonia.

## DISCLOSURE

Publication charges were funded by IdISSC. No other potential conflict of interest relevant to this article was reported.

## Notes

KEY POINTS **QUESTION:** What is the association between metabolic and structural changes in lung parenchyma in asymptomatic cancer patients with suspected COVID-19 pneumonia? **PERTINENT FINDINGS:** The SUV_max_ of lung parenchyma infiltrates is closely associated with several tomographic changes characteristic of COVID-19 pneumonia (CO-RADS categories) in asymptomatic cancer patients undergoing ^18^F-FDG PET/CT. **IMPLICATIONS FOR PATIENT CARE:** ^18^F-FDG PET/CT may provide additional information about early diagnosis of COVID-19 pneumonia in cancer patients during the pandemic.
